# Supplementary material for: A new species of Allodaposuchus (Eusuchia, Crocodylia) from the Maastrichtian (Late Cretaceous) of Spain: phylogenetic and paleobiological implications
Source: PeerJ. 2015 Aug 13;3:e1171. doi: 10.7717/peerj.1171 (PMC4558081; doi:10.7717/peerj.1171)
Supplement: Supplemental Information S1 [file peerj-03-1171-s001.docx]

**Supporting information S1**

Phylogenetic relationships of *Allodaposuchus hulki* were explored using the dataset of Brochu (2011). However modifications in some operational taxonomic units (OTUs) and characters were carried out.

According to Delfino *et al.* (2008), we added a new state to the characters 148 and 149 (102 and 132 in Delfino *et al.* [2008]). In both cases, the state 0 is assigned to *Hylaeochampsa*, *Allodaposuchus* and *Arenysuchus*. In the rest of taxa from the original matrix of Brochu (2011) the state 0 passes to be 1 and the state 1 becomes 2 (for the description of the new state of character see Delfino *et al.* [2008]).

In the present study, *Allodaposuchus precedens* from the original matrix of Brochu (2011) was replaced by the codification of the specimen (PSMUBB V 438) from Oarda de Jos (Romania) (see Delfino *et al.*, (2008) for details). Our coding for *A*. *precedens* is the same as in Delfino *et al.* (2008) but the characters were reordered according to Brochu (2011). Seventeen new characters were coded to *A*. *precedens* according to the figures and the description of Delfino *et al.* (2008): 19 (?), 20 (?), 80 (0), 84 (?), 85 (0), 105 (?), 106 (0), 107 (0), 131 (0), 141 (0), 142 (0), 148 (0), 149 (0), 157 (0), 178 (0), 179 (0), 180 (0). In addition, the taxa *Arenysuchus gascabadiolorum* and *Allodaposuchus subjuniperus* and *Allodaposuchus palustris* were added to the matrix (Puértolas-Pascual et al., 2011; 2013; Blanco et al., 2014; respectively). Two characters were also modified to *A. subjuniperus* according to the descriptions of Puértolas-Pascual *et al.* (2013): 87 (1), 174 (0).

The character state codings for *A. hulki* are:

*Allodaposuchus hulki:* ?????????? ??????1??0 1??101?101 ?????0???? ??????11?? ?????????? ?????????? ????????00 0?0?00101? ?????????? ?1??0?0??? ?????????? ?????????? ????1?10?1 00?1100000 111021010? ?????10??? ???0??1000 0

**References**

1. Blanco, A., Puértolas-Pascual, E., Marmi, J., Vila, B., Sellés, A.G., 2014. *Allodaposuchus palustris* sp. nov. from the upper Cretaceous of Fumanya (south-eastern Pyrenees, Iberian Peninsula): systematics, palaeoecology and palaeobiogeography of the enigmatic allodaposuchian crocodylians. PLoS ONE
2. Brochu CA (2011) Phylogenetic relationships of *Necrosuchus ionensis* Simpson, 1937 and the early history of caimanines. Zool J Linn Soc 163: S228-S256.
3. Delfino M, Codrea V, Folie A, Dica P, Godefroit P, et al. (2008) A complete skull of *Allodaposuchus precedens* Nopcsa, 1928 (Eusuchia) and a reassessment of the morphology of the taxon based on the Romanian remains. J Vert Paleontol 28: 111-122.
4. Puértolas E, Canudo JI, Cruzado-Caballero P (2011) A new Crocodylian from the Late Maastrichtian of Spain: Implications for the initial radiation of crocodyloids. PLoS ONE (6, 6) e20011. doi:10.1371/journal.pone.0020011
5. Puértolas-Pascual E, Canudo JI, Moreno-Azanza M (2013) The eusuchian crocodylomorph *Allodaposuchus subjuniperus* sp. nov., a new species from the latest Cretaceous (upper Maastrichtian) of Spain. Historical Biol. doi:10.1080/08912963.2012.763034
