# Supplementary material for: A new species of Allodaposuchus (Eusuchia, Crocodylia) from the Maastrichtian (Late Cretaceous) of Spain: phylogenetic and paleobiological implications
Source: PeerJ. 2015 Aug 13;3:e1171. doi: 10.7717/peerj.1171 (PMC4558081; doi:10.7717/peerj.1171)
Supplement: Supplemental Information S2 [file peerj-03-1171-s002.doc]

nstates 16 ;

xread 'Data saved from TNT'

182 85

Bernissartia_fagesii ???????0???0111102100?00?0?000???0000?100010???0010?000???????10?0?00?001?1????000?0?0000?00030?001?????1000????1?0000?000??0100?0???000100?0?0??0?0?1010?0??00??0????????000?0000?000

Acynodon_adriaticus ??????1?????????????01?100?1??????????010?10?????????01????????0???0??100??00??00010?000?0?10600001?0????110????00?00011010000?1?111?0?0100???0????0??010?0?0010??????????????1???01?0

Acynodon_iberoccitan ???????????????????????????????????????????????10104101????????0???????0?0?????00010?0000001060000100??00100????0000000101?00???200100?0100?110?0000??010100000?1?????????1???0????010

Iharkutosuchus_makad ???????????????????????????????????????????????10124?????????110???00??10?1????0001??00000110610001?0???0110????00000010011000012011001?100?1?0?00?0???12???100?2?????1???1000000??110

Hylaeochampsa_vectia ?????????????????????????????????????????????????????????????????????????0?????0??????????0?0?10001?00??0110?0?00000001001000?0?21110000120????1?0?000010100000000????1?001001000?0110

Borealosuchus_forabi ?000?000?0?11001001001000000101000001?000?20???0110200000?000?110000000100001??00020?0000?00231000100?00000010000000101001000101002001?11000000?00?011010100101000??????00100110000000

Borealosuchus_wilsoi ???????0??????????1001000000101??00?1?000?20???01002??100?0?001100000201000????00020?0?0???02310001?????00001?0?0?0010100100010100200101100?000100??111101001010000?00???010011?0?0000

Borealosuchus_acutid ?????????????????????????????0???????????????????002????????????000??????0?????00020?0?00??02310001?????0000????0?0??0???????1??0020?1?1100?0?0?0??0?111010?101000???????010??1?0?0000

Borealosuchus_sternb ?000000000?110010?1001000000101000001?00???0???011020000000?00010000000100000??00020?000000013100010001000001?0?000001110100010100000111100?000100?011000100101000000?1?00100110000000

Eothoracosaurus_miss ???????0????????????01??000????????00?00???0????122???3??????0?????00?011?0????00120?000??1025000010??000000????0000101001000????000?000100???01???11100010?001?00????????100010000000

Thoracosaurus_neoces ???????0??????111?1?010????0011??0??0?00???0???1122???3????????10?000?01?0?????00120?0000?10250000100??000000?0?00001010010000?000000000110?00010??1?100010000100000000?00100?10000000

Eosuchus_minor ???????0???0??111???01?00?0?01???0000?000??0???1122??0300?0000?10?000001100????00120?0000?1025?000100?000000?00000001010010000?100000000110???0100?11?100100001?0100??1???101010101003

Eogavialis_africanum ?????????1?????11???010??????????0?????????0???1122??03?????10?100000??1101????00120?000?01025?000100?0000000000000010100100000100000000121?000100?1111001000010100?000??0101010100000

Gryposuchus_colombia ?????0?0???001??????01??000????????????????0???11223?030100?000100000001100????0012010000?1025?000100?0?0000??0?000010100100000110000000121?000100?11111010000200000100?00121010100000

Gavialis_gangeticus ?020000000?001111011010000000111000000?0000000011223003000000001000000011000100001301000001025?000100000000000000000101001000001100000001210000100011111010000100000000000121010100000

Pristichampsus_vorax ?????0?0???01001001?01?00000111??0100?10???1???1110?000?????0??1000001?1?0?????21010?0000?00030001100?000000??0?000000100100010100000111110???0100?021000100101000???01??1100110000002

Pristichampsus_geise ??????????????0?0???01?000?0??1??0100?1???1????11102000??????0?1????0??1?1?????21?20?00000000300011???000000????0000001001010??100000111110?0?0?00?0?110010?10100?????????1???10000002

Planocrania_hengdong ?????????????????????1?????????????????????????1110???1????????1????0?01?0?????20010?0?????01300??1????0?000????0????01001???????0?0?1?1100????????0?1?00100?0100?????1???1???10?10001

Planocrania_datangen ???????????????????????????????????????????????11?????0????????????????????????20010?000??10030000100??0?000???????000??01???????0?0?1?1110????????0?100010010??0?????????????10??0?0?

Leidyosuchus_canaden ?????0?0???????1????010000011?1??10?0?11??11???0110?00000?0?01?100000011101????00010?000000003000010000010001000100001110100010100010111100?010100101200010010100000001?00100110010001

Diplocynodon_ratelii ???????0??????000???010?00?1111001400?10??21???01002101?????0111000001?1101????00120?00000012300001000101000??0?000000111100010100010111100??10100101100010110100000001?10100110010001

Diplocynodon_hantoni ?100???1?1?01000010001000011111??1400?101?21???011021010????011100000111101????00120?0?000?11300?0100?1010001?0?000000101100010100010111100?110100?012010101101000??0?1??010011?010001

Diplocynodon_mueller ?????????????????????1?01??1?????14?0?10??21???01002??2?????01110??10011100????00120?10000012300001000101000????00000111110000010001?1?1100?110??0?02?01010110100????????0100110010001

Diplocynodon_tormis ?????????????????????1?????1??????????10??21???01?021?1?????01?1?00????11??????0?120??000001230000100?1010001???000000111100?0?100010111100?110100001201010110100?0???1?001?0110010001

Diplocynodon_darwini ?100001001?010000?00010000?1111??1400?101121???011020010??0?01?10000011110100??00020?0000?01030000100?0??000??0?0000001111?001010001?111100?110100?022010101101000???????0100110010001

Baryphracta_deponiae ?100?0?0???????0????01?0???1?????14?0?10??21????1?02??0?????01?10??0??1110?????001???000??010300001?0????000?????0?0001?11000?010001?111100?110100?0??011?0?101?00????????1???1??10001

Stangerochampsa_mcca ?????110???010010?0001000001111001000?01??11???111010100????0111110000?110?????00110?0002?11020000100?001000????000000011110010120011111100?110200?0?2100102101000??1?1??0100110010001

Albertochampsa_langs ???????????????????????????????????????????????????????????????????????????????001?0?0?0??110200001?0??01?00????00??00011110010?2001?111100???0?0??0?210010210100????????010011001?000

Brachychampsa_montan ?101011001?1100???0001??000111100?000?103111???11101101?????01110?00001110100??00110?0002?11010000100?0010001?0?010000011110010120011111100?110200?02210010110102000101?00100110010001

Brachychampsa_sealey ??????????????????????????????????????10???1???11101??0???????11??0000111?1????001?0?0002?1101000010???010?????????00????????10?200?1101?00???????????100???????2?????????????1??10??1

Alligator_sinensis ?101011101?110010100010110111110011000112111110110000120?001011200000111101??110010000100001020000100000100011001000000111100101101111111100110200102220010210101000111100100110010001

Alligator_mississipp ?101011001?010010000010110111110011000112101100110001120100101120001011110100110010000100001020000100000100011100000000111100101101111111100110200102221010210101000111100100110010001

Alligator_mefferdi ????????????????????????????1?????????11???????110000120100001120001011110100??00100?0100?01020000100?0?10001?1?0000000?1110010110111111110?110200?022210102101010??1???00100110010001

Alligator_thomsoni ????????????????????01????????1?????0?1????????110000?2?????011200010011101????00100?010000102000910??0010001?1?000000????1?????10111111110?1?0200102?21010210101?001?11?0100110010001

Alligator_olseni ???????0?1?????10???01?10011111???100?11???????11010010?????011200000011101????00100?01000?10200??100???1000??0?0?0001011111010110111111110?110200?022200102101010??????00100?1?010001

Alligator_mcgrewi ?100010001?010010?00010000111?1101??0?11???1???11110010?100?011100000111101????00000?0100?01020000100?0010001?001000000111100101101111111000110200?022200102101010?01???00100110010001

Alligator_prenasalis ?10001?0?1?????10?0?01000011111??1000?112111???11111010?????01110000011110100??00000?0100?01020000100?0010001100100000011110010110111111100?110200?022200102101000001?1?00100110010001

Ceratosuchus_burdosh ????????????????????????????????????0?1????????11111??0?????01?10??00?11?01????00010?0?01?01020000100???1000??0????00???01???1?1??0??111100????????0????010?111???????????10??10010001

Hassiacosuchus_haupt ?001?1?0?1??????0???01?000?1111?????0??11?11???111110?0?????01?10??0??11101????00010?00???010?00001??????0001?0??????0?????????11001?111100?1?0200?02220010210101????????01???10010001

Navajosuchus_mooki ???????0?1??????0???0?00???1111??1??0?111111???11111010?????01?10??00??110?????00010?0001?010200001???0?1000??0???00?00?1110010110011111100???020??0222?0102101000????1??010??10010001

Allognathosuchus_pol ???????????????????????????????????????????????11111010?????01?11??00?111?1????00010?000??010200001?0?????00??0?00?00?0?111001?1?011?111100????????0??20010?101???????????1???1??1???1

Allognathosuchus_war ?????1?0????????????0?0000?1111??1000?11???1???11111010?100?011110000011101????00010?0000?0102000010000010001?0?000000?0111001?110111111100?110200?0222001021010000?1?1?0010011001?001

Wannaganosuchus_brac ?????1?0???1?00?0???010000?1111001000?11???1???111110?0?????0??100?00?11?0?????00110?0000??10200??100???1000??0?100000???1??01?11011?111100????????0?22001??101000????????10011?010001

Procaimanoidea_kayi ?????110?1??????0???010?00?1111??10?0?112121?????010?1??100?01?11000001110?????10?????0???010?00001???0?1000??0?0000000011100?01101111111000??0200?022200102101010???????0100110010001

Procaimanoidea_utahe ???????????????????????????????????????????????110100?00??01011110??0011101????10110?0100?01020?00100??01000????0000000?111001011011?111100?1?0200?02220010?101000???????0100110010001

Arambourgia_gaudryi ???????????????????????????????????????????????11010??0?????01?100?0??1110?????1001??010??010200001?0???10001000??000????11?0??11011?111100?1102?0?0?2210?0210100?????????10011?010?01

Necrosuchus_ionensis ???????0????????????0???01?111???1300?11???1???1100???2????????1???0?????0?????00????????????????????????????????????????????????????????????????????????????????0??????????0????1?0?1

Tsoabichi_greenriver ????????????????????01????????????????10??20???1100???2????????1??????11???01??00010?10????10??0001????????0????????????????????0001?1111?0?1?0????0??211???101?2????????????????100??

Purussaurus_mirandai ?????????????????????1?11001?????11?0?1????????1100?0?2??1?11?1201100?11?01????0?110?0001101020010100???1000????0000000111101?11111??111110?1102001022201???10102??????????0011?2?00?1

Purussaurus_neivensi ?101?100?1?000010?0??1????011???????0?11???1???1?00??1?1010?111201100011001????00110?0001?01020000100?0010001?0?0?00000111101211111?1111110?110201?0222011?210102000101??010??10210001

Orthogenysuchus_olse ?????????????????????????????????????????????????00??????????????????????0?????00121?0?01??10?0?0?1??????000????01?000????????????????11110????????0????11??101??0???????????????10001

Mourasuchus_spp ?10??100?1?00?010?00?1?10?011????1300?11???1???1102?112?????01110?100011000????00121?0000?11050001100?0010001?0?01000001111012?1011??111110?110?00????2111??111?30????1???100110?10001

Eocaiman_cavernensis ???????????????????????????????????????????????1110???2?????11?1???????????????00????0??????0?????1?????100???0?1??0000???????0??????1??1?0?????0???????0?????1?3??????????0??1?2?????

Caiman_yacare ?101111001?100010000010101111110011000111221110110021121010111010110201100101110011000000001120000100000100011001000000111101211110111111100110201102220111210103000101100100110210001

Caiman_crocodilus ?101111001?100010000010101111110011000111221110110021121010111010110201100101110011000000001120000100000100011001000000111101211100111111100110201102220111210103000101100100110210001

Caiman_latirostris ?101110001?10001000001010?111110011000111221210110021121010111?10110201100???110011000000001020010100000100011001000000111101211100111111100110201102221111210103000101100100110210001

Caiman_lutescens ?????????????????????????????????????????????????0?????????????????????????????00110?0000?01020010100?001000????1000000111101211200??1?1?10?????0???????1????0???????????????????100?1

Melanosuchus_fisheri ???????0????????0?????????????????????????????????02????????1?11011????10?1????001?0?0000??1020010110???1000??0???0????11????2?11????111110????????0????11??1010?????????010??1?2?0001

Melanosuchus_niger ?101111001?1?00100000101011111100110001112212101100211210101111101102011001??110011000000001020010110000100011001000000111101211100111111100110201102221111210103000101100100110210001

Paleosuchus_trigonat ?100111111?01001010001000111111211300011132112011002122211111111011000110010111101100001000102000010000010001100010001011110111100011111110111020110222111?210102000101100100110210001

Paleosuchus_palpebro ?100111111?010010101010001111112113000111321120110021222111?11?10110001100?0111101100001000102000010000010001100010001011110111100011111110111020110222111?210102000101100100110210001

Mecistops_cataphract ?10?001001?000010000011100111112012000111101101111041010100010010001110101?10010012000000100210000100010000011010000101101000101000001111100001011101220010010100011101011110010000003

Crocodylus_niloticus ?101000001?101010001011100111112012000111201101110021010100010110001110101110010011000000100210000100110000011010010001101000101100001111100001011101220010010100011101011110011000003

Crocodylus_porosus ?111000001?001010101011100011112012000111201101110021010100010110001110101110010011000000100210001100110000011010010001101000101000001111100001011101220010010100011101011110011000003

Crocosylus_acutus ?001000001?101011001011100111112012000110201101110021010100010110001110101110010011000000100210100100110000011010010001101000101100001111100001011101220010010100011101011110011000003

Osteolaemus_tetraspi ???1?00001?001010100011100111112011100111111101110021010100010110000110101110011010000010100210001100010000011011001011101010101000101111101001010101221110010110011101011110110000003

Osteolaemus_osborni ???1?00001?001010100011100111112011100111111101110021010100000110000110101110011011000010100210001100010000011011001001001010101000001111101001010101221110010110011101011110110000003

Voay_robustus ???????0????????0???011?????111??1110??????1???1110210101000001100011111011????00110?0000?0021000110001000001101000101110101010100000111110?0010111012201100111110?1101?11110010000003

Rimasuchus_lloydi ??????????????????????????????????????????????????????1??????????????????1?????00110?0000?00210001100?100000110100000????10??1??1000?111110?001011?012200100101100111?1??1110?1000?003

Crocodylus_megarhinu ???????0???????????001?????????????????????????11102101?????00110000??01011????00110?0000?0023000010001?0000??0?000000110100010120000111110?002?11?0122?01001010?01100???1110010000003

Australosuchus_clark ???????0???????1??0??1?????11???????0?10???1???1110?101?????001100001101011????00110?000011021000010001000001?0?000000???????101000?0111110?002011?02220010010100011??1??11?001000?001

Kambara_implexidens ???????0????????????01?????11????1100?10???1???11102101?????001100001101011????00110?000011021000010001000001?01000000100100010100000111110?002011?01220010010100011101?11110010000001

Trilophosuchus_rackh ???????????????????????????????????????????????????????????????????????????????0?????0??????2?0??0??0?1000????0?1?000001010???01?0??0111110??12011102221010000102011101?111?0?1000?001

Tomistoma_schlegelii ?021000001?0010100010110001111110110001013011011122?1040000010010000000101000010012000000110210000101010000011010001001001000101100001111100000110101221010010100011001011110010000003

Thecachampsa_america ???????????????1????01?000?1111??1100?00???1???1122???4?????00010?0031?1010????00120?0000110210000100?100000????0000100001000???1000?011110?000?10?11220010010101???0????11100100?2003

Kentisuchus_spenceri ???????0????????????????????????????????????????1?????0?????0??1000?1101?11????00110?0000?10210000100???0000??0?010010100100?1011000?111110??????0?0??20010?10100???????111?0?100?0?03

Crocodylus_acer ???????????????????????????????????????????????????????????????????????????????00110?0000?10210000100??000001???000100?10100010100010111100?002?01?01?2001001010001??0??11100010000003

Crocodylus_affinis ?001001001?10001000011100001111001100?10???1???111021010100000110000000101100??00110?0000100110000100?010000??0?000100110100010100000111100?0?010??0122001001010001??0???1100010000003

Asiatosuchus_germani ?001?0?0?1?001010?0101?000?1111??1??0??????1???11102000?????00110000??0101?00??00010?0000?0001000010???10000??0?00010???010001010000?111100???0100?012110100101000???????1100?10000003

Prodiplocynodon_lang ???????????????????????????????????????????????????????????????????????????????00110?0000??0030000100??100001??000010011?1000101???0?111100???0?0??0?21?010?1010001??01?01100110000003

Arenysuchus_gascabad ???????????????????????????????????????????????????????????????????????????????00010?00?0000??00001???0?0?000???0?0??011010000?110000101110???01?000??01100?101???0???10?01000??0?000?

Allodaposuchus_subju ???????????????????????????????????????????????????????????????????????????????00110?00100?0?20000100?0000000???00010011010000???00?01?1110?000100000001100?1010??????1???10000?010000

Allodaposuchus_prece ???????????????????????????????????????????????????????????????????????????????00010?0000101220000100?100?00????00000?1??10?00???00001?1110??001?0000001000?10100?????0?101?100101000?

Allodaposuchus_palus ???????????0100100??01???????????1000??????????110????1??????????????????0?????00??????????????????????????0?????????????????????0?0?????1?????????00000110?1010??????????1???1?011000

Coco_CasaFaba ?????????????????1??01??101?101?????0??????????11??????????????????????????????000?0?00101????????????1??0?0???????????????????????????1?10?100?1100000111021010??????10??????0??10000

;

Ccode

-[/1 0 -[/1 1 -[/1 2 -[/1 3 -[/1 4

-[/1 5 -[/1 6 -[/1 7 -[/1 8 -[/1 9

-[/1 10 -[/1 11 -[/1 12 -[/1 13 -[/1 14

-[/1 15 -[/1 16 -[/1 17 -[/1 18 -[/1 19

-[/1 20 -[/1 21 -[/1 22 -[/1 23 -[/1 24

-[/1 25 -[/1 26 -[/1 27 -[/1 28 -[/1 29

-[/1 30 -[/1 31 -[/1 32 -[/1 33 -[/1 34

-[/1 35 -[/1 36 -[/1 37 -[/1 38 -[/1 39

-[/1 40 -[/1 41 -[/1 42 -[/1 43 -[/1 44

-[/1 45 -[/1 46 -[/1 47 -[/1 48 -[/1 49

-[/1 50 -[/1 51 -[/1 52 -[/1 53 -[/1 54

-[/1 55 -[/1 56 -[/1 57 -[/1 58 -[/1 59

-[/1 60 -[/1 61 -[/1 62 -[/1 63 -[/1 64

-[/1 65 -[/1 66 -[/1 67 -[/1 68 -[/1 69

-[/1 70 -[/1 71 -[/1 72 -[/1 73 -[/1 74

-[/1 75 -[/1 76 -[/1 77 -[/1 78 -[/1 79

-[/1 80 -[/1 81 -[/1 82 -[/1 83 -[/1 84

-[/1 85 -[/1 86 -[/1 87 -[/1 88 -[/1 89

-[/1 90 -[/1 91 -[/1 92 -[/1 93 -[/1 94

-[/1 95 -[/1 96 -[/1 97 -[/1 98 -[/1 99

-[/1 100 -[/1 101 -[/1 102 -[/1 103 -[/1 104

-[/1 105 -[/1 106 -[/1 107 -[/1 108 -[/1 109

-[/1 110 -[/1 111 -[/1 112 -[/1 113 -[/1 114

-[/1 115 -[/1 116 -[/1 117 -[/1 118 -[/1 119

-[/1 120 -[/1 121 -[/1 122 -[/1 123 -[/1 124

-[/1 125 -[/1 126 -[/1 127 -[/1 128 -[/1 129

-[/1 130 -[/1 131 -[/1 132 -[/1 133 -[/1 134

-[/1 135 -[/1 136 -[/1 137 -[/1 138 -[/1 139

-[/1 140 -[/1 141 -[/1 142 -[/1 143 -[/1 144

-[/1 145 -[/1 146 -[/1 147 -[/1 148 -[/1 149

-[/1 150 -[/1 151 -[/1 152 -[/1 153 -[/1 154

-[/1 155 -[/1 156 -[/1 157 -[/1 158 -[/1 159

-[/1 160 -[/1 161 -[/1 162 -[/1 163 -[/1 164

-[/1 165 -[/1 166 -[/1 167 -[/1 168 -[/1 169

-[/1 170 -[/1 171 -[/1 172 -[/1 173 -[/1 174

-[/1 175 -[/1 176 -[/1 177 -[/1 178 -[/1 179

-[/1 180 -[/1 181 ;

Ancstates

-0 -1 -2 -3 -4 -5 -6 -7 -8 -9

-10 -11 -12 -13 -14 -15 -16 -17 -18 -19

-20 -21 -22 -23 -24 -25 -26 -27 -28 -29

-30 -31 -32 -33 -34 -35 -36 -37 -38 -39

-40 -41 -42 -43 -44 -45 -46 -47 -48 -49

-50 -51 -52 -53 -54 -55 -56 -57 -58 -59

-60 -61 -62 -63 -64 -65 -66 -67 -68 -69

-70 -71 -72 -73 -74 -75 -76 -77 -78 -79

-80 -81 -82 -83 -84 -85 -86 -87 -88 -89

-90 -91 -92 -93 -94 -95 -96 -97 -98 -99

-100 -101 -102 -103 -104 -105 -106 -107 -108 -109

-110 -111 -112 -113 -114 -115 -116 -117 -118 -119

-120 -121 -122 -123 -124 -125 -126 -127 -128 -129

-130 -131 -132 -133 -134 -135 -136 -137 -138 -139

-140 -141 -142 -143 -144 -145 -146 -147 -148 -149

-150 -151 -152 -153 -154 -155 -156 -157 -158 -159

-160 -161 -162 -163 -164 -165 -166 -167 -168 -169

-170 -171 -172 -173 -174 -175 -176 -177 -178 -179

-180 -181 ;

xgroup

;

agroup

;

taxcode

+0 +1 +2 +3 +4 +5 +6 +7

+8 +9 +10 +11 +12 +13 +14 +15

+16 +17 +18 +19 +20 +21 +22 +23

+24 +25 +26 +27 +28 +29 +30 +31

+32 +33 +34 +35 +36 +37 +38 +39

+40 +41 +42 +43 +44 +45 +46 +47

+48 +49 +50 +51 +52 +53 +54 +55

+56 +57 +58 +59 +60 +61 +62 +63

+64 +65 +66 +67 +68 +69 +70 +71

+72 +73 +74 +75 +76 +77 +78 +79

+80 +81 +82 +83 +84

;

blocks 0;

proc/;
